# Muscle function assessed by the non-invasive method acoustic myography (AMG) in a Danish group of healthy adults

**DOI:** 10.1016/j.crphys.2020.02.002

**Published:** 2020-02-12

**Authors:** Else Marie Bartels, Jack Kvistgaard Olsen, Eva Littrup Andersen, Bente Danneskiold-Samsøe, Henning Bliddal, Lars Erik Kristensen, Adrian P. Harrison

**Affiliations:** a The Parker Institute, Copenhagen University Hospital, Bispebjerg and Frederiksberg, Denmark; b Department of Neurology, Copenhagen University Hospital, Bispebjerg and Frederiksberg, Denmark; c Copenhagen Center for Translational Research, Copenhagen University Hospital, Bispebjerg and Frederiksberg, Denmark; d Department of Clinical Medicine, Faculty of Health and Medical Sciences, Copenhagen University, Denmark; e PAS (Physiology), Faculty of Health & Medical Sciences, Copenhagen University, Denmark

**Keywords:** Acoustic myography, Sonography, Muscle fatigue, Activities of daily living

## Abstract

Acoustic myography (AMG) is a non-invasive method to assess muscle function during daily activities. AMG has great scope for assessment of musculoskeletal problems. The aim of this study was to create an AMG data set for general clinical use and relate these findings to age and gender. 10 healthy subjects (5 men/5 women), in each decade from 20 to 69 years of age (*n* = 50), were assessed. Their clinical health was tested. AMG measurements were carried out on muscles involved in defined movements of the upper and lower extremities. Muscle performance was measured using efficiency (E-score) and fibre recruitment (temporal (T-score) and spatial (S-score) summation). AMG-measurements showed good reproducibility. In each age group, it was found that for all those daily living skills measured, there was no gender difference. A walking and stair climbing test revealed that both legs are used equally and in a balanced way in healthy subjects. Moreover, there was no change in this function with increasing age up to 69 years. However, a cycling test with loading revealed that in elderly subjects the coordination of muscle use is impaired compared to that of the younger adults. Finally, a flexion test of the arm revealed an age-related decrease in the efficiency/coordination of m.Biceps alone, and a keyboard writing test suggests no effect on m.Trapezius. This reference data set now illustrates the reproducibility and ease of use of acoustic myography in the clinic and provides a means of assessing individuals with musculoskeletal problems.

## Highlights

• Acoustic myography is a non-invasive, real-time and repeatable tool for assessing musculoskeletal problems in the clinic.
• A walking and stair climbing test revealed both legs to be used in a balanced way in healthy subjects, with no age effect.
• A cycling test with loading showed that coordination of muscle use was impaired with increasing age.
• A flexion test of the arm revealed an age-related decrease in the efficiency/coordination of m.Biceps alone.
• This reference data set illustrates the ease of use of this technique in the clinic for musculoskeletal problems.

## Graphical abstract

**Figure:**
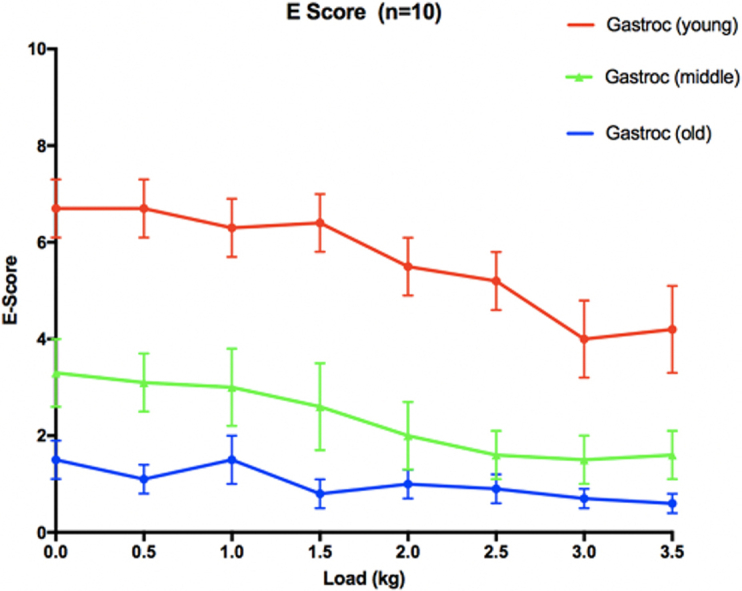

## 1 Background

Quality of life is closely related to independence when carrying out the most common activities of daily living (Imagama et al., 2017; Jayakaran, Perry, & Hale, 2019). Non-invasive measurements of muscle function during daily activities are therefore important in the general assessment of patients with pain and/or functional problems. They are also valuable when assessing athletes, particularly when looking at the effects of training. For all subjects, efficient use of muscles when wishing to carry out tasks over a longer time span, for example walking or cycling, is of great importance. This is especially true when decreasing muscle strength due to illness or age occurs (Danneskiold-Samsoe et al., 2009), or when professional athletes wish to reach excellency (Bartels et al., 2017; Vesterinen et al., 2016). Until recently, surface Electromyography (sEMG) (de Luca, 1997; Hermens et al., 1999) has been the main successful non-invasive method in laboratory settings (Bernardi et al., 1999; Gerdle, Karlsson, Crenshaw, & Friden, 1997; Harrison, Mølsted, Pingel, Langberg, & Bartels, 2012, chap. 5; Karlsson & Gerdle, 2001; Larsson, Kadi, Lindvall, & Gerdle, 2006), often requiring a shielded laboratory or a noise free setting, and the difficulty in correctly interpreting the data due to a combined neuromuscular signal (Hermens et al., 1999), results in limitations which cannot be easily overlooked (Harrison, 2018).

The gold-standard sEMG technique has been assessed in human subjects alongside acoustic myography (AMG), a technique that was developed some years ago (Harrison, Danneskiold-Samsoe, & Bartels, 2013). As has been documented for sEMG, acoustic myography is also non-invasive, reliable and easily applicable (Barry, Geiringer, & Ball, 1985). A further advantage of AMG is an easier data handling, which is also more precise, and has the possibility of measuring over several hours and in normal daily settings outside the laboratory (Bartels et al., 2017; Harrison, 2018; Harrison et al., 2013). It has been shown with AMG that force production is regulated in a similar and age-independent way in men and women by changing the spatial and/or temporal summation of the muscle fibres in a muscle, indicating a clear recruitment pattern (Claudel, Ahmed, Elbrond, Harrison, & Bartels, 2018). It is well accepted that muscle force is the product of a change in the number of active motor units (spatial summation), as well as a change in the rate of repetitive firing of such active motor units (temporal summation) (Dideriksen, Negro, Enoka, & Farina, 2012). The reproducibility of AMG measurements is, furthermore, in canine experiments seen to be excellent (Fenger & Harrison, 2017). The method has also been demonstrated to enable assessment of development of muscle fatigue during muscle use (Barry et al., 1985, 1992; Bartels et al., 2017; Claudel et al., 2018; Fenger & Harrison, 2017). In this respect fatigue was assessed by a loss of spatial summation and temporal summation, as well as a loss of coordination during periods of sustained muscle work, all three of which are parameters that are measured using AMG (Claudel et al., 2018; Harrison, 2018). In general, this technique provides information of the slightest difference in the use of left/right sides of the body e.g. left and right legs for gait assessment (walking). As a technique, it also shows the muscle effort and coordination associated with daily movement related tasks. This in itself is very useful in the rheumatology clinic or with neuromuscular subjects (Harrison, 2018).

At present, AMG reference data on healthy subjects and for common movements is not available, making its application restricted for general use in the clinic.

The aim of this study was to create such a set of reference data for the AMG parameters expressed in the ESTi™ Score (Harrison, 2018). These parameters express muscle fibre use in a measured muscle during a defined movement. The data of selected important muscles during defined movements has been collected from a group of healthy adults aged 20–69 years, giving the opportunity to look for possible age and gender differences, and reproducibility of measurements on this human cohort has also been tested. It is our intention that these data will form the base for clinical measurements of patients with musculoskeletal difficulties and for athletes for training purposes, as well as during rehabilitation.

## 2 Methods

### 2.1 Ethics

The study followed the guidelines set by the Helsinki Declaration 2013 (http://www.wma.net/en/30publications/10policies/b3/), and the subjects were asked to give an informed written consent prior to participation in the study. The AMG method applied was non-invasive, so the only invasive procedure was a standard blood test. The study was approved by the Capital Region of Denmark's Ethics Committee (H-15017787) and was registered with the Danish Data Protection Agency.

All data were handled according to The Act on Processing of Personal Data, May 2018.

This study was part of a larger study looking at different aspects of muscle condition and function in a healthy Danish population.

### 2.2 Subjects

50 healthy subjects took part in the study. 5 men and 5 women in each age decade from 20 to 69 years of age were included following recruitment via advertising or via word of mouth. The number of subjects was decided based on a Power calculation on previously published data (Claudel et al., 2018; Pingel et al., 2019), revealing a power of approximately 80% for this number of subjects in each age group. All measurements were carried out at the Parker Institute, Copenhagen University Hospital, Bispebjerg and Frederiksberg, Denmark. Since the participants were healthy, blinding towards groups was not relevant. Staff fully trained in the use of the AMG setup carried out the measurements. Data handling was blinded.

Inclusion criteria were:

• Age 20–69 years of age
• 18.5 < BMI <30 (BMI = Body Mass Index, measured in kg/m^2^)
• Being healthy according to an examination and a standard blood test (see Table 1) prior to measurements

**Table 1:** A list of the blood test parameters included for the assessment of the participants as being healthy individuals prior to participation in the study. The results were controlled by a medical doctor and a specialist technician to ensure that values were within the normal range for healthy subjects as per the University Hospitals in the Capital Region (www.bispebjerghospital.dk/).

1	Glycated Haemoglobin-c (HbA1c)
2	Alanine-transaminase
3	Alkaline Phosphatase
4	C-Reactive Protein (CRP)
5	Erythrocytes
6	Erythrocyte volume fraction (EVOL)
7	Erythrocyte volume Mean (MCV)
8	Haemoglobin (Hb)
9	Mean corpuscular haemoglobin concentration (MCHC)
10	Potassium
11	Creatinine
12	Leucocytes
13	Leucocyte type and group (DIFFMAS)
14	Sodium
15	Reticulocyte group
16	Thrombocytes
• Reporting no chronic or no present illness
• No intake of medicine, except birth control pills
• No caffeine containing drinks were consumed up to 2 h before the experiment start
• No alcohol was consumed up to 12 h before the experiment start
• Pain reported in the normal range and pattern for healthy subjects, according to the PainDETECT Questionnaire (PD-Q) ©2005Pfizer Pharma GmbH (Freynhagen, Baron, Gockel, & Tolle, 2006), prior to participation

Exclusion criteria were:

• Younger than 20 or older than 69 years of age
• No understanding of the Danish language

The routine blood test was performed on all of the subjects, identical to that used when assessing patients for musculoskeletal diseases at Copenhagen University Hospital, Bispebjerg and Frederiksberg. For details of the parameters tested see Table 1.

### 2.3 Acoustic myography (AMG)

#### 2.3.1 Equipment

AMG was recorded with a CURO unit (sampling rate 2 kb/s) and CURO sensors (CURO-Diagnostics ApS, Frederiksberg, Denmark). Measurements were followed by the CURO Clinic Data recording system, showing ESTi score and real-time recordings on an iPadAir (Apple Inc, Cupertino, CA, USA). The sensors had a frequency recording range of 0.5–20 ± 0.5 kHz. Data stored on a SD card within the CURO unit was transferred to the CURO software for calculation of the ESTi-score, describing muscle performance by efficiency (E-score) and fibre recruitment temporal (T-score) and spatial (S-score) summation. These terms refer to spatial- and temporal-summation of the motor units (Harrison, 2018). It should be noted that the E, S and T-scores are without units (Harrison, 2018). In brief, the E -score corresponds to the periods of active compared to inactive muscle function, expressed in terms of the duration of the active period of the muscle (in other words how long the muscle is “on”), the S -score reflects the recruitment of motor units and equates to signal amplitude (how many motor units are active), and the T -score is the motor unit firing rate or signal frequency (how fast the motor units are firing) (Harrison, 2018). With these parameters, an intuitive assessment of optimal muscle function, based on a scale of 0–10 was adopted, where 0 was considered as 0% optimal and 10 was considered 100% optimal. To calculate the scores, the measured mV amplitude, for example, was subtracted from the maximal mV amplitude that could be accurately detected. The difference was then divided by the maximal amplitude and multiplied by 10 to yield a 0–10 scoring system.

#### 2.3.2 Measurements

Measurements were taken from the lower extremities from *m. Gastrocnemius* and *m. Rectus femoris*, from the upper extremities from *m. Biceps* and *m. Triceps*, from the upper back from *m. Trapezius*, and from the hand *m. Abductor pollicis brevis*. The sensors were covered with ultrasound gel (EKO Gel, EkkoMed, Holstebro, Denmark) and taped to the skin with bandage (Mediplast AB, Malmö, Sweden) above the central part of the muscle body. Sensor positioning has been shown to be less critical than that of sEMG, and so long as one places a sensor on the body of a muscle, it will provide comparable results (Harrison, 2018). For a detailed figure of the AMG signal recorded during a period of increasing cycling intensity, as well as a comparison of a typical sEMG and AMG signal see Harrison (2018).

The following movements and their associated muscle groups were measured, and in the precise order listed below. Indeed, such movements as walking, cycling, climbing stairs and arm movements, were adopted since they represent activities that everyone needs in order to routinely carry our daily living.

1) Walking 1 min (AMG: *m. Gastrocnemius*, both legs). Repeat of the 1-min walk.
2) Stair walking >30 s, 16 steps up and 16 steps down (AMG: *m. Gastrocnemius*, both legs) repeated in duplicate.
3) Passive and active bending of arm (AMG: *m.Biceps* and *m.Triceps*)
4) 45 min measurements of Isokinetic muscle strength, upper and lower extremities. Data from these measurements will be reported in a separate paper due to it being a very different form of muscle assessment with a need of different data handling and setting up of the standard data for further use in the clinic.
5) Thumb: Flexion/extension and touching little finger with thumb (*m. Abductor pollicis brevis*)
6) Cycling test at increasing load from low load to as high as the subject could accept (typically 0.0–3.5 kg; 20 ± 2 km/h; 125 ± 10 Watts: Monark ergomedic 874E bicycle). (AMG: *m. Rectus femoris* and *m. Gastrocnemius*) measured on the subject's dominant leg. (speed)
7) Writing on keyboard for 1 min (AMG: *m. Trapezius*). The reason for this measurement is a claim from office workers that they experience pain in their shoulders during computer work (Ishikawa et al., 2017), hence the choice of m.Trapezius that is involved in the rotation, retraction, elevation and depression of the scapula, as well as levitation of the clavicle.

It should be noted that the tests listed above were chosen because they are standard tests for patients with musculoskeletal diseases at the Frederiksberg campus, Copenhagen University Hospital. Furthermore, between each sequence 1) to 7) the subjects rested for 5 min. The exercises above were carried out at the subject's normal and comfortable speed, as we were interested in how recruitment of muscles takes place in healthy subjects with increasing age, and not in a comparison of the maximal force produced by each subject (i.e. we were not measuring force).

### 2.4 Reproducibility

The criteria used to assess the reproducibility of the AMG technique was the delta value between the first and second set of ESTi measurements, where an acceptable value of 5% was adopted. Indeed, it was found that a maximum divergence between the first and second measurements never exceeded 0.3 (3% of the ESTi-score).

### 2.5 Data handling

Data was analysed by the CURO data handling programme (CURO-Diagnostics ApS, Copenhagen, DK), giving the ESTi™-score with its individual components Efficiency (E-score) and fibre recruitment (Temporal (T-score) and Spatial (S-score) summation), which are all mean values from the recorded activity trace. Furthermore, the ST score, which is the average value of the two, was calculated. This score is based on the fact that the CNS can either change spatial summation and/or change temporal summation when producing an increase in force, such that different individuals use a different combination of the two, yet ultimately reach the same result (Claudel et al., 2018).

When looking at changes in age, gender or load and their interactions, differences between means showing a *P* value > 0.05 were considered non-significant. Data were tested for normality and equal variance. Due to the study design, in which it has been necessary to examine more than two parameters simultaneously, an ANOVA analysis was adopted. A 2-way ANOVA accommodating multiple comparisons was performed using the statistical software of Prism 8 (Version 8.3.0 for macOS, GraphPad Software LLC). Where interactions between row factors (e.g. age) and column factors (e.g. load) were examined using a Tukey's multiple comparisons test.

### 2.6 Study sequence

1) Information about the study given, and written information sent/given in hand with consent form. Written consent signed prior to participation in study.
2) Visit including:
  a) Answering PainDetect Questionnaire and general health questionnaire concerning medicine and chronic disease
  b) Blood tests and weight and height measurements
  c) Assessment of fulfilling the inclusion criteria
  d) AMG measurements

## 3 Results

### 3.1 Subjects

The essential parameters for the subjects that participated in this study are presented in table form (see Table 2).

**Table 2:** A description of the healthy population participating in this study. All values are given as mean ± standard deviation (SD). Weight is given in kilograms (kg) and height in meters (m).

Age Group	Men				Women			
	Age (years)	Weight (kg)	Height (m)	BMI (kg/m^2^)	Age (years)	Weight (kg)	Height (m)	BMI (kg/m^2^)
20–29 years	28 ± 1	90 ± 1	1.86 ± 0.07	26.0 ± 1.7	25 ± 4	64 ± 1	1.71 ± 0.06	21.8 ± 1.1
30–39 years	35 ± 3	90 ± 11	1.85 ± 0.04	26.0 ± 2.8	35 ± 5	63 ± 6	1.69 ± 0.07	22.0 ± 4.3
40–49 years	44 ± 5	90 ± 8	1.83 ± 0.01	26.8 ± 2.2	44 ± 3	62 ± 1	1.65 ± 0.06	22.7 ± 2.0
50–59 years	53 ± 3	83 ± 19	1.81 ± 0.10	25.5 ± 3.2	54 ± 2	67 ± 11	1.69 ± 0.04	23.4 ± 4.1
60–69 years	65 ± 3	72 ± 8	1.76 ± 0.03	23.3 ± 2.7	62 ± 2	69 ± 18	1.65 ± 0.03	25.4 ± 5.4

### 3.2 Reproducibility

The sequences of walking were repeated (1 & 2) without any rest period between the two repeats, and measurements for *m. Gastrocnemius* for the left leg and right leg were analyzed for reproducibility.

The reproducibility of the ESTi scores for the left leg was found to be excellent. When comparing the two measurements (sequence 1 and sequence 2), a delta (SD) between each individual subject's E, S and T parameters showed an overall value of 0.0 (E), 0.1 (S) and 0.0 (T), with a value of 0.1 for the average of the S and T parameters combined (*n* = 48; 2 data sets missing due to recording error).

The reproducibility of the ESTi scores for the right leg was found to be excellent. When comparing the two measurements (sequence 1 and sequence 2), a delta between each individual subject's E, S and T parameters showed an overall value of 0.0 (E), 0.1 (S) and 0.1 (T), with a value of 0.0 for the average of the S and T parameters combined (*n* = 48; 2 data sets missing due to recording error).

The reproducibility of the ESTi scores for the left leg when ascending stairs was found to be excellent. When comparing the two measurements (sequence 1 and sequence 2), a delta between each individual subject's E, S and T parameters showed an overall value of 0.0 (E), 0.1 (S) and 0.2 (T), with a value of 0.2 for the average of the S and T parameters combined (*n* = 48; 2 data sets missing due to recording error).

The reproducibility of the ESTi scores for the right leg when ascending stairs was found to be excellent. When comparing the two measurements (sequence 1 and sequence 2), a delta between each individual subject's E, S and T parameters showed an overall value of 0.0 (E), 0.0 (S) and 0.2 (T), with a value of 0.1 for the average of the S and T parameters combined (*n* = 48; 2 data sets missing due to recording error).

The reproducibility of the ESTi scores for the left leg when descending stairs was found to be excellent. When comparing the two measurements (sequence 1 and sequence 2), a delta between each individual subject's E, S and T parameters showed an overall value of 0.0 (E), 0.1 (S) and 0.2 (T), with a value of 0.2 for the average of the S and T parameters combined (*n* = 48; 2 data sets missing due to recording error).

The reproducibility of the ESTi scores for the right leg when descending stairs was found to be excellent. When comparing the two measurements (sequence 1 and sequence 2), a delta between each individual subject's E, S and T parameters showed an overall value of 0.0 (E), 0.3 (S) and 0.2 (T), with a value of 0.2 for the average of the S and T parameters combined (*n* = 48; 2 data sets missing due to recording error).

An ANOVA (see Appendix 2) confirmed these results. Furthermore, a more detailed analysis showed no difference in the score values for this muscle and type of activity between males and females.

### 3.3 Age differences

As a representative muscle for the lower extremities, the *m. Gastrocnemius* during walking, showed an E, S, T and ST score for the 5 age-groups as shown in Table 3. As a representative muscle for the upper extremities, the *m. Biceps* during active flexion and extension, showed an E, S, T and ST score for the 5 age-groups as shown in Table 3. As a representative muscle for the non-extremities, the *m.Trapezius* during writing, showed an E, S, T and ST score for the 5 age-groups as shown in Table 3. Based on that there were only 5 men and 5 women in each age group, and the reproducibility test for *m. Gastrocnemius* showed no significant effect of gender, it was decided to pool data for men and women in each age group.

**Table 3:** The E, S, T and ST scores for three representative muscles over age (mean ± SD). A lower extremity muscle (*m. Gastrocnemius*), an upper extremity muscle (*m. Biceps*) and a non-extremity muscle (*m. Trapezius*) are presented.

*m.Gastrocnemius*	E	S	T	ST
20–29 years	0.9 ± 0.7	2.1 ± 1.1	5.9 ± 0.9	4.0 ± 0.5
30–39 years	2.0 ± 0.7	0.6 ± 1.0	2.3 ± 1.9	3.7 ± 0.8
40–49 years	0.6 ± 0.5	2.3 ± 1.4	5.9 ± 1.3	4.1 ± 1.0
50–59 years	0.7 ± 0.4	2.1 ± 1.4	6.7 ± 1.1	4.4 ± 0.8
60–69 years	0.6 ± 0.5	0.9 ± 0.8	7.8 ± 0.7	4.4 ± 0.6
*m. Biceps*				
20–29 years	5.9 ± 2.4	7.7 ± 1.6	5.7 ± 2.5	6.7 ± 0.1
30–39 years	5.5 ± 1.6	7.7 ± 0.7	4.3 ± 2.6	6.0 ± 0.1
40–49 years	4.4 ± 2.2	8.0 ± 1.0	5.2 ± 2.6	6.7 ± 0.1
50–59 years	4.5 ± 2.6	7.6 ± 1.3	4.7 ± 2.0	6.1 ± 0.1
60–69 years	2.5 ± 1.1	7.3 ± 2.2	3.8 ± 1.4	5.6 ± 0.1
*m.Trapezius*				
20–29 years	9.1 ± 1.3	9.8 ± 0.1	7.2 ± 0.7	8.5 ± 0.4
30–39 years	9.5 ± 0.5	9.7 ± 0.1	6.6 ± 2.3	8.2 ± 1.2
40–49 years	9.6 ± 0.4	9.7 ± 0.2	7.0 ± 2.6	8.3 ± 1.4
50–59 years	9.5 ± 0.4	9.7 ± 0.2	7.7 ± 2.5	8.6 ± 1.3
60–69 years	9.4 ± 0.9	9.8 ± 0.1	7.4 ± 2.1	8.7 ± 1.1

An ANOVA (see Appendix 2) revealed that for the men there was only one small yet significant difference between the 40 and 50 year groups and only for the right leg *m. Gastrocnemius* T-score, which is most likely to be a type-2 error. However, for the women a more consistent pattern was found, for the left leg *m. Gastrocnemius* the T-score was significantly different between the 20–59 year olds and the oldest group.

### 3.4 Gender differences

Although no gender differences were found above, we did decide to analyse the pooled data from all of the men with the pooled data from all of the women in order to confirm our earlier findings (see reproducibility). Pooling these data is, furthermore, a valid action since no age effect was noticed. An ANOVA (see Appendix 2) confirmed these results.

As a representative muscle for the lower extremities, the *m. Gastrocnemius* during walking, showed an overall E-score (mean ± SD) for the females of 0.8 ± 0.3 and for the males it was 0.6 ± 0.1. The overall S-score for the females was 2.0 ± 0.6 and for the males it was 1.8 ± 0.7. The overall T-score for the females was 6.2 ± 1.2 and for the males it was 6.5 ± 0.8. Finally, the overall ST-score for the females was 4.1 ± 0.3 and for the males it was 4.2 ± 0.3.

As a representative muscle for the upper extremities, the *m. Biceps* during active flexion and extension showed an overall E-score for the females of 4.3 ± 1.0 and for the males it was 4.4 ± 1.5. The overall S-score for the females was 7.5 ± 0.9 and for the males it was 7.3 ± 0.7. The overall T-score for the females was 4.0 ± 0.6 and for the males it was 5.1 ± 1.4. Finally, the overall ST-score for the females was 5.8 ± 0.4 and for the males it was 6.2 ± 0.9.

As a representative muscle for the non-extremities, the *m. Trapezius* during writing showed an overall E-score for the females of 9.4 ± 0.3 and for the males it was 9.3 ± 0.3. The overall S-score for the females was 9.6 ± 0.1 and for the males it was 9.8 ± 0.1. The overall T-score for the females was 6.7 ± 1.4 and for the males it was 8.3 ± 0.6. Finally, the overall ST-score for the females was 8.2 ± 0.6 and for the males it was 9.1 ± 0.3. No significant difference was found for gender, confirming our earlier findings (Claudel et al., 2018).

### 3.5 Walking on a level surface–balance

The data measured for *m. Gastrocnemius* during walk on a flat hard surface, are presented for both the left and the right legs (see Table 4). They not only show a very consistent AMG score with age, but also a very even balance between the left and right legs.

**Table 4:** AMG-data for *m. Gastrocnemius* left and right when walking on a flat surface (given as mean ± SD).

*m.Gastrocnemius*	E Left/Right	S Left/Right	T Left/Right	ST Left/Right
20–29 years	0.9 ± 0.7/1.0 ± 0.8	2.1 ± 1.1/2.6 ± 1.9	5.9 ± 0.9/6.4 ± 1.1	4.0 ± 0.5/4.5 ± 1.0
30–39 years *	2.0 ± 0.7/2.2 ± 0.8	0.6 ± 1.0/0.6 ± 1.5	2.3 ± 0.9/2.3 ± 1.3	3.7 ± 0.8/4.1 ± 0.9
40–49 years	0.6 ± 0.5/0.4 ± 0.4	2.3 ± 1.4/1.4 ± 1.1	5.9 ± 1.3/6.2 ± 1.0	4.1 ± 1.0/3.8 ± 0.9
50–59 years *	0.7 ± 0.4/0.8 ± 0.3	2.2 ± 1.3/1.8 ± 0.9	6.7 ± 1.1/5.6 ± 2.2	4.4 ± 0.8/3.7 ± 1.1
60–69 years	0.6 ± 0.5/0.9 ± 0.9	0.9 ± 0.8/1.5 ± 0.8	7.8 ± 0.7/7.7 ± 0.7	4.4 ± 0.6/4.6 ± 0.5

Each group represents the mean of 20 values (5 men and 5 women measured twice), except for the * groups where one individual is missing.

### 3.6 Stairs up and down–balance

The data measured for *m. Gastrocnemius* during ascending stairs are presented for both the left and the right legs (see Table 5, Table 6). Again, these data reveal a very balanced and consistent use of both the left and right leg muscles in this healthy population when ascending a flight of stairs. It is also clear that no age-related differences exist for this type of movement. An ANOVA (see Appendix 2) showed that for both men and women there was a significant difference between the T-score for *m. Gastrocnemius* between the youngest group and the oldest, when ascending and descending stairs.

**Table 5:** AMG data for *m. Gastrocnemius* during ascending stairs (given as mean ± SD).

*m.Gastrocnemius*	E Left/Right	S Left/Right	T Left/Right	ST Left/Right
20–29 years	1.3 ± 0.8/1.8 ± 1.1	3.1 ± 1.2/4.0 ± 1.4	5.3 ± 1.6/5.1 ± 1.6	4.2 ± 1.3/4.6 ± 1.3
30–39 years	2.4 ± 0.7/2.0 ± 0.7	0.6 ± 1.3/0.6 ± 1.6	2.3 ± 1.4/2.3 ± 1.4	4.1 ± 1.0/3.9 ± 1.2
40–49 years	1.2 ± 0.7/1.0 ± 0.8	3.3 ± 1.1/2.7 ± 1.6	5.7 ± 1.6/5.6 ± 1.9	4.5 ± 0.7/4.2 ± 1.3
50–59 years *	1.3 ± 0.8/1.7 ± 1.7	2.9 ± 1.8/3.0 ± 1.5	6.0 ± 1.5/6.2 ± 1.8	4.6 ± 0.9/4.7 ± 0.9
60–69 years	1.1 ± 0.8/1.0 ± 0.7	2.0 ± 1.0/2.1 ± 1.0	6.8 ± 1.2/6.3 ± 1.3	4.4 ± 0.7/4.2 ± 0.8

Each group represents the mean of 20 values (5 men and 5 women measured twice), except for the * groups where one individual is missing.

**Table 6:** AMG data for *m. Gastrocnemius* during descending stairs (given as mean ± SD).

*m.Gastrocnemius*	E Left/Right	S Left/Right	T Left/Right	ST Left/Right
20–29 years	1.3 ± 0.7/1.6 ± 0.9	1.8 ± 1.1/2.5 ± 1.4	6.0 ± 1.8/6.2 ± 1.4	3.9 ± 1.0/4.3 ± 0.7
30–39 years *	2.4 ± 0.7/2.4 ± 0.7	0.6 ± 1.2/0.6 ± 1.9	2.3 ± 2.3/2.3 ± 1.8	3.2 ± 1.2/4.1 ± 0.9
0–49 years #	1.0 ± 0.7/0.8 ± 0.6	1.4 ± 0.7/1.7 ± 1.3	5.7 ± 1.9/5.8 ± 1.4	3.6 ± 1.0/3.7 ± 1.0
50–59 years *	0.8 ± 0.5/1.3 ± 1.6	2.5 ± 1.5/2.5 ± 2.1	6.6 ± 1.0/6.5 ± 1.1	4.5 ± 0.7/4.5 ± 1.2
60–69 years **	0.6 ± 0.4/0.9 ± 0.7	2.5 ± 0.8/2.3 ± 1.1	7.1 ± 1.2/7.8 ± 0.8	4.8 ± 0.7/5.0 ± 0.4

Each group represents the mean of 20 values (5 men and 5 women measured twice), except for the * groups where one individual is missing (left leg) and ** 3 missing from the left leg. # indicates 1 missing from the right leg.

The data measured for *m. Gastrocnemius* during descending a flight of stairs, are presented for both the left and the right legs. There are no signs of age differences, nor any signs of preferential use of either the right or left leg.

### 3.7 Cycling with increasing load

**Fig. 1:**
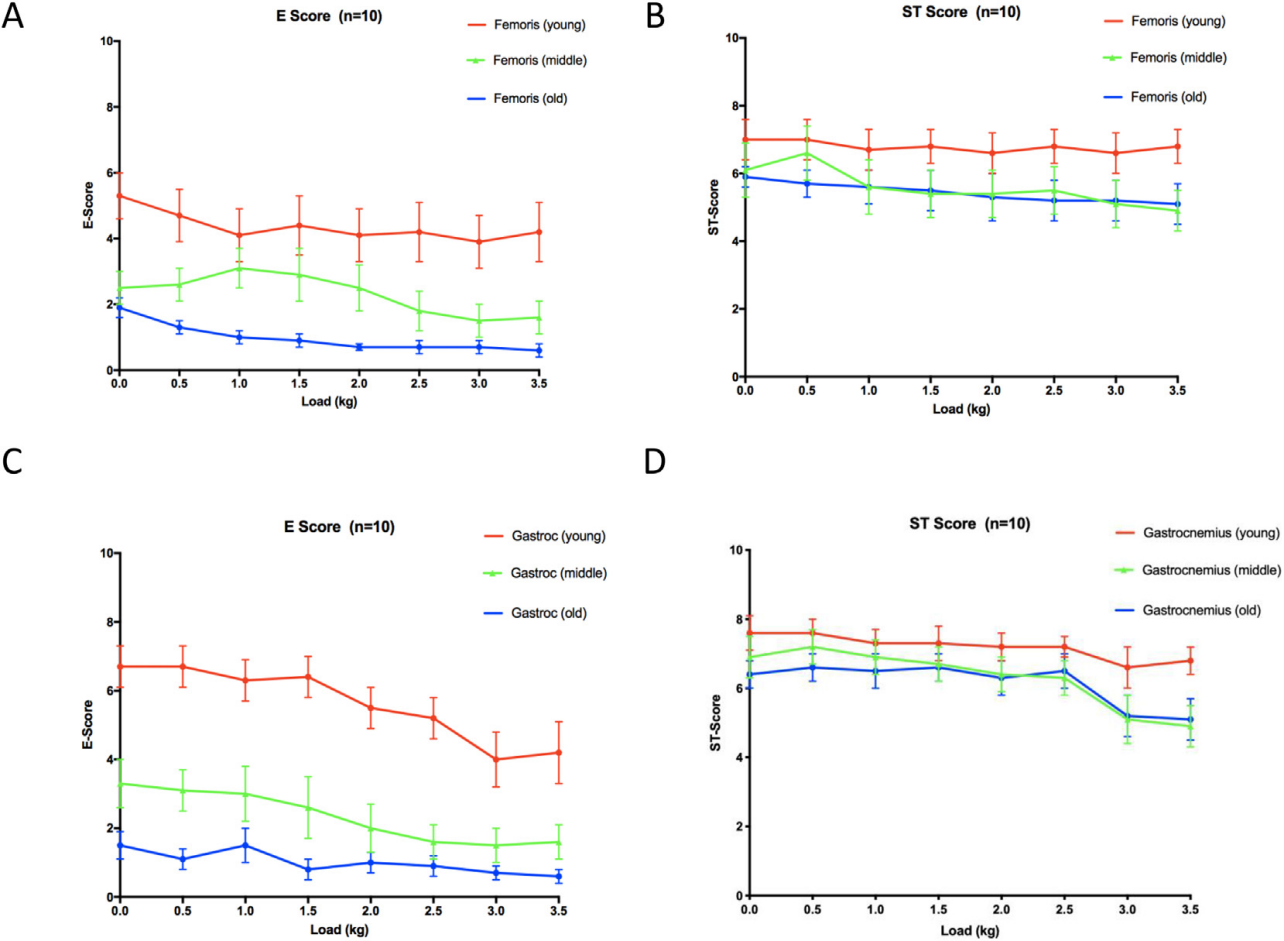
Presents the E-score and ST-scores for healthy individuals spanning the age range of 20–69 years. Younger subjects (20–29 years) are shown in red. The middle-aged group (40–49 years) are shown in green, and the older group (60–69 years) are represented by the blue symbols. Subjects cycled at a set speed of 20 ± 2 km/h and 125 ± 10 Watts. Panels a and b represent *m. Femoris*, whilst panels c and d represent *m. Gastrocnemius*. The E-score and the ST-score for *m. Femoris* were both found to be significantly different between the younger and the older groups; *P* < .001. Likewise, the E-score and the ST-score for *m. Gastrocnemius* were also both found to be significantly different between the younger and the older groups; *P* < .001. Values are the mean ± SEM.

The data for *m. Femoris* (see Fig. 1a and b) and *m. Gastrocnemius* (see Fig. 1c and d) for the dominant leg of the subjects in this study are shown for cycling with increasing load. For the sake of clarity we have chosen to show the youngest, oldest and middle aged groups only for the cycling data obtained.

As seen from the graphs (Fig. 1), there is a clear and significant age effect on the E-score (*P* < 0.001) and to a lesser extent the ST-score (*P* < 0.001) for both muscles measured in the cycle test. It is interesting to note that the changes begin to appear from an age of 40 years and onwards (middle group). This coincides with changes in proprioception (Lord, Delbaere, & Sturnieks, 2018) whereby the degree of accuracy concerning ROM and joint positioning begins to become affected from an age of 40 years. An ANOVA (see Appendix 2) confirmed the statistical differences seen in Fig. 1.

### 3.8 Flexion test–active

The data measured for *m. Biceps* and *m. Triceps* during voluntary flexion and extension, are presented for the subject's dominant arm (see Table 7, Table 8). This shows that the E-score is age dependent for *m. Biceps*, indicative of a lower degree of efficiency/coordination for this movement with increasing age (lower E-score), whilst the other parameters remain very balanced and consistent, albeit with a greater degree of individual variation (Standard Deviation). An ANOVA (see Appendix 2) for *m. Biceps* revealed a significant difference between the four younger groups and the oldest group for men and the E-score alone. For women this parameter was only significantly different between the 50′s and 60′s age groups. Whilst for *m.Triceps* there were no significant differences for either gender.

**Table 7:** AMG data for *m. Biceps* during flexion (given as mean ± SD).

*m.Biceps*	E	S	T	ST
20–29 years	5.9 ± 2.4	7.7 ± 1.6	5.7 ± 2.5	6.7 ± 2.1
30–39 years	5.8 ± 1.4	7.6 ± 0.7	4.4 ± 2.7	6.0 ± 1.7
40–49 years	4.4 ± 2.5	7.9 ± 1.0	5.2 ± 2.6	6.6 ± 1.8
50–59 years	4.5 ± 2.6	7.6 ± 1.3	4.6 ± 1.9	6.1 ± 1.6
60–69 years	2.4 ± 1.1	7.4 ± 2.2	3.8 ± 1.4	5.6 ± 1.8

However, the same observation cannot be concluded for *m. Triceps*.

**Table 8:** AMG data for *m. Triceps* during flexion (given as mean ± SD).

*m.Triceps*	E	S	T	ST
20–29 years	6.9 ± 2.1	8.0 ± 1.6	4.6 ± 2.1	6.3 ± 1.9
30–39 years	6.8 ± 1.5	8.5 ± 0.7	3.2 ± 2.0	5.0 ± 1.4
40–49 years	6.3 ± 1.8	7.5 ± 1.5	5.9 ± 2.5	6.7 ± 2.0
50–59 years	7.1 ± 1.7	8.7 ± 0.9	5.6 ± 3.1	7.2 ± 1.9
60–69 years	4.4 ± 2.4	8.8 ± 0.5	4.8 ± 2.3	6.8 ± 1.4

The data for passive flexion-extension can be seen in Appendix 1. This does not differ significantly from the voluntary movement in healthy subjects.

### 3.9 Writing test

The data for *m. Trapezius* for the left and right side of the subjects, during a standard keyboard writing test, are shown below (see Table 9). They show no difference between left and right sides, and in general this muscle is used very slightly in these healthy subjects, as noted by the very high E, S and T-scores. An ANOVA (see Appendix 2) confirmed these results.

**Table 9:** AMG data for *m. Trapezius* during a keyboard writing test (given as mean ± SD).

*m.Trapezius*	E Left/Right	S Left/Right	T Left/Right	ST Left/Right
20–29 years	9.0 ± 1.3/9.1 ± 0.6	9.8 ± 0.1/9.6 ± 0.1	7.2 ± 0.7/7.6 ± 1.6	8.5 ± 0.4/8.6 ± 0.9
30–39 years	9.5 ± 0.5/9.2 ± 0.6	9.7 ± 0.1/9.7 ± 0.1	6.6 ± 2.3/7.0 ± 1.8	8.2 ± 1.2/8.4 ± 1.1
40–49 years	9.6 ± 0.3/9.5 ± 0.6	9.7 ± 0.2/9.4 ± 0.4	7.0 ± 2.6/6.5 ± 3.0	8.3 ± 1.4/8.0 ± 1.7
50–59 years	9.4 ± 0.4/9.3 ± 0.4	9.6 ± 0.2/9.7 ± 0.2	8.7 ± 0.7/8.2 ± 1.1	9.2 ± 0.5/8.9 ± 0.6
60–69 years	9.4 ± 0.9/8.8 ± 0.9	9.8 ± 0.1/9.8 ± 0.1	7.5 ± 2.3/8.2 ± 2.4	8.6 ± 1.2/8.9 ± 1.3

### 3.10 Thumb test

The data for *m. Abductor pollicis brevis* during flexion and extension of the thumb are shown below (see Table 10). There appears to be no obvious effect of age on this movement. An ANOVA (see Appendix 2) confirmed these results.

**Table 10:** AMG data from *m. Abductor pollicis brevis* during flexion and extension of the thumb (given as mean ± SD).

*m.Abductor pollicis brevis*	E	S	T	ST
20–29 years	6.5 ± 2.4	8.4 ± 1.2	4.7 ± 2.5	6.6 ± 0.0
30–39 years	6.8 ± 1.6	8.4 ± 1.4	4.8 ± 2.4	6.6 ± 0.0
40–49 years	8.6 ± 2.0	9.1 ± 0.9	5.8 ± 2.6	7.5 ± 0.0
50–59 years	6.0 ± 3.1	7.7 ± 2.5	4.1 ± 3.1	5.9 ± 2.4
60–69 years	6.1 ± 2.9	8.6 ± 0.9	5.2 ± 3.1	6.9 ± 0.0

The data for m. Abductor pollicis brevis during the thumb test involving thumb and little finger touching, are shown below (see Table 11). It appears that with increasing age, the ST parameter decreases slightly, suggesting that more fibres and/or a higher firing frequency is necessitated for this type of movement. When looking at the ST-score, healthy individuals show very accurate thumb movements up to the age of 69.

**Table 11:** AMG data from *m. Abductor pollicis brevis* during touch test thumb to little finger (given as mean ± SD).

*m.Abductor pollicis brevis*	E	S	T	ST
20–29 years	8.0 ± 1.9	8.2 ± 1.4	5.0 ± 3.0	6.6 ± 0.0
30–39 years	5.9 ± 2.7	6.6 ± 2.0	2.3 ± 1.4	4.4 ± 0.0
40–49 years	7.2 ± 2.3	8.8 ± 0.9	3.8 ± 3.1	6.3 ± 0.0
50–59 years	5.5 ± 3.0	7.2 ± 2.5	3.5 ± 1.7	5.4 ± 0.0
60–69 years	4.6 ± 2.9	7.2 ± 2.0	3.8 ± 2.2	5.5 ± 0.0

## 4 Discussion

These collated tests represent the first acoustic myography data for healthy individuals, covering the age-range of the working life of most adults. They show quite clearly that there are age-related changes in the AMG measurements. Furthermore, these results represent a number of typical physical tasks which are important for assessing subjects in terms of their ability to carry out daily living skills. The data presented reveal that acoustic myography as a technique is very repeatable based on the outcomes of the daily living skill tests in this study, for which repeat measurements were made. This makes the method applicable for use in the clinic.

In each age group, it was found that for all those daily living skills measured, there was no gender difference. For this reason, the data sets for each age group were pooled. These data are also in good accordance with an earlier study, in which a human fore-arm muscle (*m. palmaris longus*) was tested (Claudel et al., 2018), and in which it was shown that the mechanisms of fibre recruitment were identical between sexes, even though ultimate maximal force differed.

It is known that force production and its maintenance by a muscle is a component of both spatial and temporal summation (Contessa, de Luca, & Kline, 2016; Dideriksen et al., 2012). An individual's approach can therefore be to either use more fibres and a low firing frequency or few fibres and a high firing frequency in order to attain the same level of force production (Claudel et al., 2018). For this very reason, the collated data from this study also presents the combined spatial and temporal signals as an ST-score, since this is a useful and valid measure for fibre use during force production. The E-score is also a very useful value since it expresses the level of efficiency/coordination with which a muscle is used (Bartels et al., 2017). The present findings that the E-score decreases with increasing age, starting in the 40–49 year group, and only in the case of physical work being applied to a muscle, is in keeping with the published findings of Lord and colleagues, in which a change in sensitivity to proprioception was noted to start in the 40–49 year old group (Lord et al., 2018). This change was documented as worsening with increasing age (Lord et al., 2018).

### Walking & stair climbing

This test resulted in very consistent values with increasing age, and comparable values for the left *versus* the right leg for all the movements, also including ascending and descending a flight of stairs. This is a useful finding since some patient groups may have difficulties in confidently completing stair walking. One might expect signs of difficulties in accomplishing this particular task as being a lower E-score and/or a lower ST-score. For subjects that have previously injured a limb and are undergoing rehabilitation, the recovery of physical function towards using both legs equally and in a balanced way can be followed using this simple AMG test during a measurement of walking.

### Cycling with load

The data clearly showed a significant effect of increasing age on the efficiency/coordination (E-score) of both *m. Femoris* and *m. Gastrocnemius* in subjects that are cycling with an increasing load (0–3.5 kg). This indicates that in elderly subjects the coordination of muscle use is impaired compared to both the middle-aged and younger adults. The reason for this change is most likely due to the known effects of ageing on proprioception (Lord et al., 2018). The main sensor in this respect is the muscle spindle receptor (mechanoreceptor) which is known to change with increasing age in terms of increased capsular thickness, decreased number per muscle, and reduced spindle diameter, all of which result in reduced spindle sensitivity in older people (Goble, Coxon, Wenderoth, Van, & Swinnen, 2009). Alternatively, an explanation may be found with age-related changes to the corpus callosum, which is known to play a role in relaying sensory and motor information between homologous regions in the two cerebral hemispheres (Ota et al., 2006).

This study also noted that the ST-score decreased with age, a change that occurred in both the middle-aged and older groups compared with the younger adults (see Fig. 1 b and d). This happens at the same time as the documented age-related reduction in the strength of involved muscles (Danneskiold-Samsoe & Bartels, 2009; Lord et al., 2018).

### Flexion test

These data reveal an age-related decrease in the efficiency/coordination of the *m. Biceps* alone. They also show a very comparable set of values for the ST-score not only with increasing age, but also between the agonist and antagonist muscles (*m. Biceps versus m. Triceps*). No change was noted for the other parameters with increasing age, nor was any significant effect noted for *m.Triceps*, although the data might suggest a tendency towards a lower E-score in the 60–69 year group. This is commensurate with the role of *m. B. versus m. Triceps* in typical arm activities for healthy subjects. The great variation in these two upper arm muscles is most likely related to the degree to which individuals use their arm muscles for daily tasks. This needs to be considered when flexion tests are used for clinical assessment. A recording from a subject showing a divergence in the ST-score between the *m. Biceps* and the *m. Triceps*, provides a means of assessing the relative harmony between these agonist/antagonist muscles during flexion.

### Writing and thumb test

This test was chosen due to the extended use of computers by most individuals in their daily life, and the risk of shoulder muscle injury as a result of sustained periods of tension (Ishikawa et al., 2017). Indeed, these authors reported an increase in stiffness of the upper trapezius is being an objective finding that may be a persistently altered condition in individuals with neck and shoulder complaints. However, in our healthy subjects we found minimal activity in this muscle group during a standard keyboard writing test. This would suggest that individuals that develop shoulder and upper back problems attributed to computer use may actually derive their muscular problems from another work environment source (Szeto, Straker, & O'Sullivan, 2009). Indeed, it was recently shown that the simple task of resting an individual's hands on a keyboard resulted in a significant increase in muscle activity in the upper *m. Trapezius* in subjects with chronic neck pain problems, compared with asymptomatic controls (Szeto et al., 2009). Using AMG during a standard keyboard writing test is therefore a useful tool to assess subjects who may later develop problems due to sustained periods of computer use, and maybe used to prevent more chronic problems later in an individual's working life.

## 5 Conclusions

The acoustic myography technique used in this study has not only proved to be quick and non-invasive, but also reproducible. It highlights muscle activity and is a potentially valuable tool in the clinic to assess an individual's ability to carry out standard daily tasks. This data set now provides reference values useable when assessing individuals with musculoskeletal problems.

## Funding

The Parker Institute was supported by core funding from the 10.13039/100001275Oak Foundation (Ocay-13-309). The project was supported by Selmont A/S and by Erna Hamiltons Fond.

## CRediT authorship contribution statement

**Else Marie Bartels:** Data curation, Writing - original draft. **Jack Kvistgaard Olsen:** Data curation. **Eva Littrup Andersen:** Data curation. **Bente Danneskiold-Samsøe:** Data curation, Writing - original draft. **Henning Bliddal:** Data curation, Writing - original draft. **Lars Erik Kristensen:** Data curation. **Adrian P. Harrison:** Writing - original draft.

## Declaration of Competing Interest

AH is in the process of establishing a company to produce and market the Acoustic MyoGraphy equipment (CURO-Diagnostics ApS), and at no time was he involved in analyzing the data. BDS's spouse is a member of the Executive Board of CURO-Diagnostics ApS, and has only a very limited number of shares in the company. BDS has no direct economic or personal interests in CURO-Diagnostics ApS, nor was she involved in the data analysis.

## Appendix A Supplementary data

Multi media component 1 Multi media component 1

Multi media component 2 Multi media component 2

## References

[bib1] Barry D.T., Geiringer S.R., Ball R.D. (1985). Acoustic myography: A noninvasive monitor of motor unit fatigue. Muscle Nerve.

[bib2] Barry D.T., Im D.J., Hill T. (1992). Muscle fatigue measured with evoked muscle vibrations. Muscle Nerve.

[bib3] Bartels E.M., Harder A., Salomons Heide K., Pingel J., Torp Andersen I., Harrison A.P. (2017). The use of acoustic myography as a measure of training effects in athletes - a 10 month case study of a BMX rider. Ann. Sports Med. Res..

[bib4] Bernardi M., Felici F., Marchetti M., Montellanico F., Piacentini M.F., Solomonow M. (1999). Force generation performance and motor unit recruitment strategy in muscles of contralateral limbs. J. Electromyogr. Kinesiol..

[bib5] Claudel C.G., Ahmed W., Elbrond V.S., Harrison A.P., Bartels E.M. (2018). The relation between maximal voluntary force in m. palmaris longus and the temporal and spatial summation of muscle fiber recruitment in human subjects. Physiol. Rep..

[bib6] Contessa P., de Luca C.J., Kline J.C. (2016). The compensatory interaction between motor unit firing behavior and muscle force during fatigue. J. Neurophysiol..

[bib7] Danneskiold-Samsoe B., Bartels E.M., Bulow P.M., Lund H., Stockmarr A., Holm C.C. (2009). Isokinetic and isometric muscle strength in a healthy population with special reference to age and gender. Acta Physiol..

[bib8] Dideriksen J.L., Negro F., Enoka R.M., Farina D. (2012). Motor unit recruitment strategies and muscle properties determine the influence of synaptic noise on force steadiness. J. Neurophysiol..

[bib9] Fenger C., Harrison A.P. (2017). The application of acoustic myography in canine muscle function and performance testing. SOJ Vet. Sci..

[bib10] Freynhagen R., Baron R., Gockel U., Tolle T.R. (2006). painDETECT: a new screening questionnaire to identify neuropathic components in patients with back pain. Curr. Med. Res. Opin..

[bib11] Gerdle B., Karlsson S., Crenshaw A.G., Friden J. (1997). The relationships between EMG and muscle morphology throughout sustained static knee extension at two submaximal force levels. Acta Physiol. Scand..

[bib12] Goble D.J., Coxon J.P., Wenderoth N., Van I.A., Swinnen S.P. (2009). Proprioceptive sensibility in the elderly: Degeneration, functional consequences and plastic-adaptive processes. Neurosci. Biobehav. Rev..

[bib13] Harrison A.P. (2018). A more precise, repeatable and diagnostic alternative to surface electromyography - an appraisal of the clinical utility of acoustic myography. Clin. Physiol. Func. Imag..

[bib14] Harrison A.P., Danneskiold-Samsoe B., Bartels E.M. (2013). Portable acoustic myography - a realistic noninvasive method for assessment of muscle activity and coordination in human subjects in most home and sports settings. Physiol. Rep..

[bib15] Harrison A.P., Mølsted S., Pingel J., Langberg H., Bartels E.M., Schwartz M. (2012). EMG methods for evaluating muscle and nerve function.

[bib16] Hermens H.J., Freriks B., Merletti R., Stegeman D., Blok J., Rau J. (1999).

[bib17] Imagama S., Hasegawa Y., Ando K., Kobayashi K., Hida T., Ito K. (2017). Staged decrease of physical ability on the locomotive syndrome risk test is related to neuropathic pain, nociceptive pain, shoulder complaints, and quality of life in middle-aged and elderly people - the utility of the locomotive syndrome risk test. Mod. Rheumatol..

[bib18] Ishikawa H., Muraki T., Morise S., Sekiguchi Y., Yamamoto N., Itoi E. (2017). Changes in stiffness of the dorsal scapular muscles before and after computer work: A comparison between individuals with and without neck and shoulder complaints. Eu. J. Appl. Physiol..

[bib19] Jayakaran P., Perry M., Hale L. (2019). Comparison of self-reported physical activity levels and quality of life between individuals with dysvascular and non-dysvascular below knee amputation: A cross-sectional study. Disabil. Health J..

[bib20] Karlsson S., Gerdle B. (2001). Mean frequency and signal amplitude of the surface EMG of the quadriceps muscles increase with increasing torque - a study using the continuous wavelet transform. J. Electromyogr. Kinesiol..

[bib21] Larsson B., Kadi F., Lindvall B., Gerdle B. (2006). Surface electromyography and peak torque of repetitive maximum isokinetic plantar flexions in relation to aspects of muscle morphology. J. Electromyogr. Kinesiol..

[bib22] Lord S.R., Delbaere K., Sturnieks D.L., Day B.L., Lord S.R. (2018). Handbook of clinical neurology.

[bib23] de Luca C.J. (1997). The use of surface electromyography in biomechanics. JAB.

[bib24] Ota M., Obata T., Akine Y., Ito H., Ikehira H., Asada T. (2006). Age-related degeneration of corpus callosum measured with diffusion tensor imaging. Neuroimage.

[bib25] Pingel J., Torp Andersen I., Broholm R., Harder A., Bartels E.M., Bülow J. (2019). An acoustic myography functional assessment of Cerebral Palsy subjects compared to healthy Controls during physical exercise. J. Muscle Res. Cell Motil..

[bib26] Szeto G.P.Y., Straker L.M., O'Sullivan P.B. (2009). Neck-shoulder muscle activity in general and task-specific resting postures of symptomatic computer users with chronic neck pain. Manual Therapy.

[bib27] Vesterinen V., Häkkinen K., Laine T., Hynynen E., Mikkola J., Nummela A. (2016). Predictors of individual adaptation to high-volume or high-intensity endurance training in recreational endurance runners. Scand. J. Med. Sci. Sport..

