# Supplementary material for: Muscle function assessed by the non-invasive method acoustic myography (AMG) in a Danish group of healthy adults
Source: Curr Res Physiol. 2020 Feb 12;2:22–9. doi: 10.1016/j.crphys.2020.02.002 (PMC8562189; doi:10.1016/j.crphys.2020.02.002)
Supplement: Multi media component 1 [file mmc1.docx]

**Appendix 1**

Table of AMG parameters for a Flexion/Extension test – passive, for both m.Biceps and m.Triceps (given as mean ± SD).

| *M.Biceps* | E  Left / Right | S  Left / Right | T  Left / Right | ST  Left / Right |
| --- | --- | --- | --- | --- |
| 20-29 years | 7.0±1.5 | 8.8±0.9 | 6.6±2.2 | 7.7±1.6 |
| 30-39 years | 6.0±2.5 | 8.8±0.6 | 5.9±2.1 | 7.4±1.3 |
| 40-49 years | 6.9±2.5 | 8.7±0.8 | 6.1±3.9 | 7.4±2.4 |
| 50-59 years | 8.2±0.5 | 9.0±0.4 | 7.5±1.9 | 8.3±1.2 |
| 60-69 years | 2.6±0.9 | 8.6±1.2 | 5.2±1.8 | 6.9±1.5 |

| *M.Triceps* | E  Left / Right | S  Left / Right | T  Left / Right | ST  Left / Right |
| --- | --- | --- | --- | --- |
| 20-29 years | 7.7±1.7 | 8.6±1.3 | 6.4±1.9 | 7.5±1.6 |
| 30-39 years | 7.1±1.2 | 9.3±0.3 | 5.6±1.5 | 7.5±0.9 |
| 40-49 years | 5.4±2.4 | 8.4±1.3 | 6.5±2.1 | 7.4±1.7 |
| 50-59 years | 8.7±0.8 | 9.2±0.6 | 8.0±2.7 | 8.6±1.7 |
| 60-69 years | 5.0±2.5 | 9.2±0.4 | 6.5±2.2 | 7.9±1.3 |
