# Supplementary material for: Muscle function assessed by the non-invasive method acoustic myography (AMG) in a Danish group of healthy adults
Source: Curr Res Physiol. 2020 Feb 12;2:22–9. doi: 10.1016/j.crphys.2020.02.002 (PMC8562189; doi:10.1016/j.crphys.2020.02.002)
Supplement: Multi media component 2 [file mmc2.docx]

**Appendix 2**

*Results of the ANOVA analyses*

WALKING – Gastrocnemius

Reproducibility

|  | E-score | S-score | T-score |
| --- | --- | --- | --- |
|  | Left / Right | Left / Right | Left / Right |
| Men 1 vs 2 | ns / ns | ns / ns | ns / ns |
| Women 1 vs 2 | ns / ns | ns / ns | ns / ns |
|  |  |  |  |
|  |  |  |  |

Men versus Women

|  | E-score | S-score | T-score |
| --- | --- | --- | --- |
|  | Left / Right | Left / Right | Left / Right |
| Men vs Women | ns / ns | ns / ns | */ ns |
| Men 2 vs Women 2 | ns / ns | ns / ns | ns / ns |
|  |  |  |  |
|  |  |  |  |

Balance between left & right

|  | E-score | S-score | T-score |
| --- | --- | --- | --- |
| Men left vs right | ns | ns | ns |
| Women left vs right | ns | ns | ns |
|  |  |  |  |
|  |  |  |  |

Age differences – with reproducibility

(1-5 refers to the different age groups)

WALK 1

|  | E-score | S-score | T-score |
| --- | --- | --- | --- |
| Men Gastro | L/R | L/R | L/R |
| 1-2 | ns | /* | ns |
| 2-3 | ns | ns | ns |
| 3-4 | ns | ns | ns |
| 4-5 | ns | ns | ns/* |
| 1-3 | ns | ns | ns |
| 1-4 | ns | ns | ns |
| 1-5 | ns | ns/* | */ns |
| 2-4 | ns | ns | ns |
| 2-5 | ns | ns | ns |
| 3-5 | ns | ns | ns |
|  |  |  |  |

|  | E-score | S-score | T-score |
| --- | --- | --- | --- |
| Women Gastro | L/R | L/R | L/R |
| 1-2 | ns | ns | ns |
| 2-3 | ns | ns | ns |
| 3-4 | ns | ns | ns |
| 4-5 | ns | ns | ***/* |
| 1-3 | ns | ns | ns |
| 1-4 | ns | ns | ns |
| 1-5 | ns | ns | ***/ns |
| 2-4 | ns | ns | ns |
| 2-5 | ns | ns | ***/ns |
| 3-5 | ns | ns | ***/ns |
|  |  |  |  |

WALK 2

|  | E-score | S-score | T-score |
| --- | --- | --- | --- |
| Men Gastro | L/R | L/R | L/R |
| 1-2 | ns | ns | ns |
| 2-3 | ns | ns | ns |
| 3-4 | ns | ns | ns |
| 4-5 | ns | ns | ns/** |
| 1-3 | ns | ns | ns |
| 1-4 | ns | ns | ns |
| 1-5 | ns | ns | */ns |
| 2-4 | ns | ns | ns |
| 2-5 | ns | ns | */ns |
| 3-5 | ns | ns | ns |
|  |  |  |  |

|  | E-score | S-score | T-score |
| --- | --- | --- | --- |
| Women Gastro | L/R | L/R | L/R |
| 1-2 | ns | ns | ns |
| 2-3 | ns | ns | ns |
| 3-4 | ns | ns | ns |
| 4-5 | ns | ns | ns |
| 1-3 | ns | ns | ns |
| 1-4 | ns | ns | ns |
| 1-5 | ns | ns | ns |
| 2-4 | ns | ns | ns |
| 2-5 | ns | ns | ***/ns |
| 3-5 | ns | ns | ***/ns |
|  |  |  |  |

STAIR CLIMBING - Gastrocnemius

ASCENDING

Reproducibility

|  | E-score | S-score | T-score |
| --- | --- | --- | --- |
|  | Left / Right | Left / Right | Left / Right |
| Men 1 vs 2 | ns / ns | ns / ns | ns / ns |
| Women 1 vs 2 | ns / ns | ns / ns | ns / ns |
|  |  |  |  |
|  |  |  |  |

Men versus Women

|  | E-score | S-score | T-score |
| --- | --- | --- | --- |
|  | Left / Right | Left / Right | Left / Right |
| Men vs Women | ns / ns | ns / ns | ns / ns |
| Men 2 vs Women 2 | ns / ns | ns / ns | ns / ns |
|  |  |  |  |
|  |  |  |  |

Balance between left & right

|  | E-score | S-score | T-score |
| --- | --- | --- | --- |
| Men left vs right | ns | ns | ns |
| Women left vs right | ns | ns | ns |
|  |  |  |  |
|  |  |  |  |

Age – with reproducibility

(1-5 refers to the different age groups)

STAIRS ASCEND 1

|  | E-score | S-score | T-score |
| --- | --- | --- | --- |
| Men Gastro | L/R | L/R | L/R |
| 1-2 | ns | ns | ns |
| 2-3 | ns | ns | ns |
| 3-4 | ns | ns | ns |
| 4-5 | ns | ns | ns |
| 1-3 | ns | ns | ns |
| 1-4 | ns | ns | ns |
| 1-5 | ns | ns | ns |
| 2-4 | ns | */ns | ns/** |
| 2-5 | ns | ns | ns/** |
| 3-5 | ns | ns | ns |
|  |  |  |  |

|  | E-score | S-score | T-score |
| --- | --- | --- | --- |
| Women Gastro | L/R | L/R | L/R |
| 1-2 | ns | ns | ns |
| 2-3 | ns | ns | ns |
| 3-4 | ns | ns | ns |
| 4-5 | ns | ns | */ns |
| 1-3 | ns | ns | ns |
| 1-4 | ns | ns | ns |
| 1-5 | ns | ns | **/ns |
| 2-4 | ns | ns | */ns |
| 2-5 | ns | ns | ***/ns |
| 3-5 | ns | ns | ns |
|  |  |  |  |

STAIRS ASCEND 2

|  | E-score | S-score | T-score |
| --- | --- | --- | --- |
| Men Gastro | L/R | L/R | L/R |
| 1-2 | ns | ns | ns |
| 2-3 | ns | ns | ns |
| 3-4 | ns | ns | ns |
| 4-5 | ns | ns | ns |
| 1-3 | ns | ns | ns |
| 1-4 | ns | ns | ns |
| 1-5 | ns | ns | ns |
| 2-4 | ns | ns | ns/* |
| 2-5 | ns | ns | ns |
| 3-5 | ns | ns | ns |
|  |  |  |  |

|  | E-score | S-score | T-score |
| --- | --- | --- | --- |
| Women Gastro | L/R | L/R | L/R |
| 1-2 | ns | ns | ns |
| 2-3 | ns | ns | ns |
| 3-4 | ns | ns | ns |
| 4-5 | ns | ns | ns |
| 1-3 | ns | ns | ns |
| 1-4 | ns | ns | ns |
| 1-5 | ns | ns | */ns |
| 2-4 | ns | ns | ns |
| 2-5 | ns | ns | **/ns |
| 3-5 | ns | ns | ns |
|  |  |  |  |

DESCENDING

Reproducibility

|  | E-score | S-score | T-score |
| --- | --- | --- | --- |
|  | Left / Right | Left / Right | Left / Right |
| Men 1 vs 2 | ns / ns | ns / ns | ns / ns |
| Women 1 vs 2 | ns / ns | ns / ns | ns / ns |
|  |  |  |  |
|  |  |  |  |

Men versus Women

|  | E-score | S-score | T-score |
| --- | --- | --- | --- |
|  | Left / Right | Left / Right | Left / Right |
| Men vs Women | ns / ns | ns / ns | ns / ns |
| Men 2 vs Women 2 | ns / ns | ns / ns | ns / ns |
|  |  |  |  |
|  |  |  |  |

Balance between left & right

|  | E-score | S-score | T-score |
| --- | --- | --- | --- |
| Men left vs right | ns | ns | ns |
| Women left vs right | ns | ns | ns |
|  |  |  |  |
|  |  |  |  |

Age – with reproducibility

(1-5 refers to the different age groups)

STAIRS DESCEND 1

|  | E-score | S-score | T-score |
| --- | --- | --- | --- |
| Men Gastro | L/R | L/R | L/R |
| 1-2 | ns | ns | ns |
| 2-3 | ns | ns | ns |
| 3-4 | ns | ns | ns |
| 4-5 | ns | ns | ns |
| 1-3 | ns | ns | ns |
| 1-4 | ns | ns | ns |
| 1-5 | ns | ns | */ns |
| 2-4 | ns | ns | ns |
| 2-5 | ns | ns | */ns |
| 3-5 | ns | ns | ns |
|  |  |  |  |

|  | E-score | S-score | T-score |
| --- | --- | --- | --- |
| Women Gastro | L/R | L/R | L/R |
| 1-2 | ns | ns | ns |
| 2-3 | ns | ns | ns |
| 3-4 | ns | ns | ns |
| 4-5 | ns | ns | ns |
| 1-3 | ns | ns | ns |
| 1-4 | ns | ns | ns |
| 1-5 | ns | ns | ns |
| 2-4 | ns | ns | ns |
| 2-5 | ns | ns | ns |
| 3-5 | ns | ns | ns/* |
|  |  |  |  |

STAIRS DESCEND 2

|  | E-score | S-score | T-score |
| --- | --- | --- | --- |
| Men Gastro | L/R | L/R | L/R |
| 1-2 | ns | ns | ns |
| 2-3 | ns | ns | ns |
| 3-4 | ns | ns | ns |
| 4-5 | ns | ns | ns |
| 1-3 | ns | ns | ns |
| 1-4 | ns | ns | ns |
| 1-5 | ns | ns | ns |
| 2-4 | ns | ns | ns |
| 2-5 | ns | ns | ns |
| 3-5 | ns | ns | ns |
|  |  |  |  |

|  | E-score | S-score | T-score |
| --- | --- | --- | --- |
| Women Gastro | L/R | L/R | L/R |
| 1-2 | ns | ns | **/ns |
| 2-3 | ns | ns | ns |
| 3-4 | ns | ns | ns |
| 4-5 | ns | ns | ns |
| 1-3 | ns | ns | ns |
| 1-4 | ns | ns | ns |
| 1-5 | ns | ns | ns |
| 2-4 | ns | ns | **/ns |
| 2-5 | ns | ns | **/* |
| 3-5 | ns | ns | ns/* |
|  |  |  |  |

CYCLING - Gastrocnemius

Men versus Women

|  | E-score | S-score | T-score |
| --- | --- | --- | --- |
|  | Gastroc | Gastroc | Gastroc |
| Men vs Women 0 | ns | ns | ns |
| Men vs Women 0.5 | ns | ns | ns |
| Men vs Women 1 | ns | ns | ns |
| Men vs Women 1.5 | ns | ns | ns |
| Men vs Women 2 | ns | ns | ns |
| Men vs Women 2.5 | ns | ns | ns |
| Men vs Women 3 | ns | ns | ns |
| Men vs Women 3.5 | ns | ns | ns |
|  |  |  |  |

Load

|  | E-score | S-score | T-score |
| --- | --- | --- | --- |
|  | Gastroc | Gastroc | Gastroc |
| Men 0 vs 0.5 | ns | ns | ns |
| Men 0 vs 1.0 | ns | ns | ns |
| Men 0 vs 1.5 | ns | ns | ns |
| Men 0 vs 2.0 | ns | ns | ns |
| Men 0 vs 2.5 | ns | ns | ns |
| Men 0 vs 3.0 | ns | ns | ns |
| Men 0 vs 3.5 | ns | ns | ns |

|  | E-score | S-score | T-score |
| --- | --- | --- | --- |
|  | Gastroc | Gastroc | Gastroc |
| Women 0 vs 0.5 | ns | ns | ns |
| Women 0 vs 1.0 | ns | ns | ns |
| Women 0 vs 1.5 | ns | ns | ns |
| Women 0 vs 2.0 | ns | ns | ns |
| Women 0 vs 2.5 | ns | ns | ns |
| Women 0 vs 3.0 | ns | ns | ns |
| Women 0 vs 3.5 | ns | ns | ns |

Age

| Load 0 | E-score | S-score | T-score |
| --- | --- | --- | --- |
|  | Gastroc | Gastroc | Gastroc |
| Men 20 vs 30 | ns | ns | ns |
| Men 30 vs 40 | ns | ns | ns |
| Men 40 vs 50 | ns | ns | ns |
| Men 50 vs 60 | ns | ns | ns |
| Men 20 vs 40 | *** | ns | ns |
| Men 20 vs 50 | ns | ns | ns |
| Men 20 vs 60 | *** | ns | ns |
| Men 30 vs 50 | ns | ns | ns |
| Men 30 vs 60 | ns | ns | ns |
| Men 40 vs 60 | ns | ns | ns |

| Load 0.5 | E-score | S-score | T-score |
| --- | --- | --- | --- |
|  | Gastroc | Gastroc | Gastroc |
| Men 20 vs 30 | ns | ns | ns |
| Men 30 vs 40 | ns | ns | ns |
| Men 40 vs 50 | ns | ns | ns |
| Men 50 vs 60 | ** | ns | ns |
| Men 20 vs 40 | ** | ns | ns |
| Men 20 vs 50 | ns | ns | ns |
| Men 20 vs 60 | *** | ns | ns |
| Men 30 vs 50 | ns | ns | ns |
| Men 30 vs 60 | ns | ns | ns |
| Men 40 vs 60 | ns | ns | ns |

| Load 1.0 | E-score | S-score | T-score |
| --- | --- | --- | --- |
|  | Gastroc | Gastroc | Gastroc |
| Men 20 vs 30 | ns | ns | ns |
| Men 30 vs 40 | ns | ns | ns |
| Men 40 vs 50 | ns | ns | ns |
| Men 50 vs 60 | ns | ns | ns |
| Men 20 vs 40 | ns | ns | ns |
| Men 20 vs 50 | ns | ns | ns |
| Men 20 vs 60 | ** | ns | ns |
| Men 30 vs 50 | ns | ns | ns |
| Men 30 vs 60 | ns | ns | ns |
| Men 40 vs 60 | ns | ns | ns |

| Load 1.5 | E-score | S-score | T-score |
| --- | --- | --- | --- |
|  | Gastroc | Gastroc | Gastroc |
| Men 20 vs 30 | ns | ns | ns |
| Men 30 vs 40 | ns | ns | ns |
| Men 40 vs 50 | ns | ns | ns |
| Men 50 vs 60 | ** | ns | ns |
| Men 20 vs 40 | *** | ns | ns |
| Men 20 vs 50 | ns | ns | ns |
| Men 20 vs 60 | *** | ns | ns |
| Men 30 vs 50 | ns | ns | ns |
| Men 30 vs 60 | ns | ns | ns |
| Men 40 vs 60 | ns | ns | ns |

| Load 2.0 | E-score | S-score | T-score |
| --- | --- | --- | --- |
|  | Gastroc | Gastroc | Gastroc |
| Men 20 vs 30 | ns | ns | ns |
| Men 30 vs 40 | ns | ns | ns |
| Men 40 vs 50 | ns | ns | ns |
| Men 50 vs 60 | ns | ns | ns |
| Men 20 vs 40 | *** | ns | ns |
| Men 20 vs 50 | ns | ns | ns |
| Men 20 vs 60 | *** | ns | ns |
| Men 30 vs 50 | ns | ns | ns |
| Men 30 vs 60 | ns | ns | ns |
| Men 40 vs 60 | ns | ns | ns |

| Load 2.5 | E-score | S-score | T-score |
| --- | --- | --- | --- |
|  | Gastroc | Gastroc | Gastroc |
| Men 20 vs 30 | ns | ns | ns |
| Men 30 vs 40 | ns | ns | ns |
| Men 40 vs 50 | ns | ns | ns |
| Men 50 vs 60 | ns | ns | ns |
| Men 20 vs 40 | ** | ns | ns |
| Men 20 vs 50 | ns | ns | ns |
| Men 20 vs 60 | *** | ns | ns |
| Men 30 vs 50 | ns | ns | ns |
| Men 30 vs 60 | ns | ns | ns |
| Men 40 vs 60 | ns | ns | ns |

| Load 3.0 | E-score | S-score | T-score |
| --- | --- | --- | --- |
|  | Gastroc | Gastroc | Gastroc |
| Men 20 vs 30 | ns | ns | ns |
| Men 30 vs 40 | ns | ns | ns |
| Men 40 vs 50 | ns | ns | ns |
| Men 50 vs 60 | ns | ns | ns |
| Men 20 vs 40 | ns | ns | ns |
| Men 20 vs 50 | ns | ns | ns |
| Men 20 vs 60 | ns | ns | ns |
| Men 30 vs 50 | ns | ns | ns |
| Men 30 vs 60 | ns | ns | ns |
| Men 40 vs 60 | ns | ns | ns |

| Load 3.5 | E-score | S-score | T-score |
| --- | --- | --- | --- |
|  | Gastroc | Gastroc | Gastroc |
| Men 20 vs 30 | ns | ns | ns |
| Men 30 vs 40 | ns | ns | ns |
| Men 40 vs 50 | ns | ns | ns |
| Men 50 vs 60 | *** | ns | ns |
| Men 20 vs 40 | ns | ns | ns |
| Men 20 vs 50 | ns | ns | ns |
| Men 20 vs 60 | ns | ns | ns |
| Men 30 vs 50 | ns | ns | ns |
| Men 30 vs 60 | ns | ns | ns |
| Men 40 vs 60 | ns | ns | ns |

| Load 0 | E-score | S-score | T-score |
| --- | --- | --- | --- |
|  | Gastroc | Gastroc | Gastroc |
| Women 20 vs 30 | ns | ns | ns |
| Women 30 vs 40 | ns | ns | ns |
| Women 40 vs 50 | ns | ns | ns |
| Women 50 vs 60 | *** | ns | ns |
| Women 20 vs 40 | ns | ns | ns |
| Women 20 vs 50 | ns | ns | ns |
| Women 20 vs 60 | *** | ns | ns |
| Women 30 vs 50 | ns | ns | ns |
| Women 30 vs 60 | ns | ns | ns |
| Women 40 vs 60 | ns | ns | ns |

| Load 0.5 | E-score | S-score | T-score |
| --- | --- | --- | --- |
|  | Gastroc | Gastroc | Gastroc |
| Women 20 vs 30 | ns | ns | ns |
| Women 30 vs 40 | ns | ns | ns |
| Women 40 vs 50 | ns | ns | ns |
| Women 50 vs 60 | *** | ns | ns |
| Women 20 vs 40 | ns | ns | ns |
| Women 20 vs 50 | ns | ns | ns |
| Women 20 vs 60 | *** | ns | ns |
| Women 30 vs 50 | ns | ns | ns |
| Women 30 vs 60 | ns | ns | ns |
| Women 40 vs 60 | ns | ns | ns |

| Load 1.0 | E-score | S-score | T-score |
| --- | --- | --- | --- |
|  | Gastroc | Gastroc | Gastroc |
| Women 20 vs 30 | ns | ns | ns |
| Women 30 vs 40 | ns | ns | ns |
| Women 40 vs 50 | ns | ns | ns |
| Women 50 vs 60 | ns | ns | ns |
| Women 20 vs 40 | ** | ns | ns |
| Women 20 vs 50 | ns | ns | ns |
| Women 20 vs 60 | *** | ns | ns |
| Women 30 vs 50 | ns | ns | ns |
| Women 30 vs 60 | ns | ns | ns |
| Women 40 vs 60 | ns | ns | ns |

| Load 1.5 | E-score | S-score | T-score |
| --- | --- | --- | --- |
|  | Gastroc | Gastroc | Gastroc |
| Women 20 vs 30 | ns | ns | ns |
| Women 30 vs 40 | ns | ns | ns |
| Women 40 vs 50 | ns | ns | ns |
| Women 50 vs 60 | *** | ns | ns |
| Women 20 vs 40 | ns | ns | ns |
| Women 20 vs 50 | ns | ns | ns |
| Women 20 vs 60 | *** | ns | ns |
| Women 30 vs 50 | ns | ns | ns |
| Women 30 vs 60 | ns | ns | ns |
| Women 40 vs 60 | ns | ns | ns |

| Load 2.0 | E-score | S-score | T-score |
| --- | --- | --- | --- |
|  | Gastroc | Gastroc | Gastroc |
| Women 20 vs 30 | ns | ns | ns |
| Women 30 vs 40 | ns | ns | ns |
| Women 40 vs 50 | ns | ns | ns |
| Women 50 vs 60 | ns | ns | ns |
| Women 20 vs 40 | ns | ns | ns |
| Women 20 vs 50 | ns | ns | ns |
| Women 20 vs 60 | ** | ns | ns |
| Women 30 vs 50 | ns | ns | ns |
| Women 30 vs 60 | ns | ns | ns |
| Women 40 vs 60 | ns | ns | ns |

| Load 2.5 | E-score | S-score | T-score |
| --- | --- | --- | --- |
|  | Gastroc | Gastroc | Gastroc |
| Women 20 vs 30 | ns | ns | ns |
| Women 30 vs 40 | ns | ns | ns |
| Women 40 vs 50 | ns | ns | ns |
| Women 50 vs 60 | ns | ns | ns |
| Women 20 vs 40 | ns | ns | ns |
| Women 20 vs 50 | ns | ns | ns |
| Women 20 vs 60 | ns | ns | ns |
| Women 30 vs 50 | ns | ns | ns |
| Women 30 vs 60 | ns | ns | ns |
| Women 40 vs 60 | ns | ns | ns |

| Load 3.0 | E-score | S-score | T-score |
| --- | --- | --- | --- |
|  | Gastroc | Gastroc | Gastroc |
| Women 20 vs 30 | ns | ns | ns |
| Women 30 vs 40 | ns | ns | ns |
| Women 40 vs 50 | *** | ns | ns |
| Women 50 vs 60 | *** | ns | ** |
| Women 20 vs 40 | ns | ns | ns |
| Women 20 vs 50 | ns | ns | ns |
| Women 20 vs 60 | ns | ns | ns |
| Women 30 vs 50 | ** | ns | ns |
| Women 30 vs 60 | ns | ns | ns |
| Women 40 vs 60 | ns | ns | ns |

| Load 3.5 | E-score | S-score | T-score |
| --- | --- | --- | --- |
|  | Gastroc | Gastroc | Gastroc |
| Women 20 vs 30 | ns | ns | ns |
| Women 30 vs 40 | ns | ns | ns |
| Women 40 vs 50 | *** | ns | ns |
| Women 50 vs 60 | *** | ns | ** |
| Women 20 vs 40 | ns | ns | ns |
| Women 20 vs 50 | ns | ns | ns |
| Women 20 vs 60 | ns | ns | ns |
| Women 30 vs 50 | ** | ns | ns |
| Women 30 vs 60 | ns | ns | ns |
| Women 40 vs 60 | ns | ns | ns |

CYCLING - Femoris

Men versus Women

|  | E-score | S-score | T-score |
| --- | --- | --- | --- |
|  | Femoris | Femoris | Femoris |
| Men vs Women 0 | ns | ns | ns |
| Men vs Women 0.5 | ns | ns | ns |
| Men vs Women 1 | ns | ns | ns |
| Men vs Women 1.5 | ns | ns | ns |
| Men vs Women 2 | ns | ns | ns |
| Men vs Women 2.5 | ns | ns | ns |
| Men vs Women 3 | ns | ns | ns |
| Men vs Women 3.5 | ns | ns | ns |
|  |  |  |  |

Load

|  | E-score | S-score | T-score |
| --- | --- | --- | --- |
|  | Femoris | Femoris | Femoris |
| Men 0 vs 0.5 | ns | ns | ns |
| Men 0 vs 1.0 | ns | ns | ns |
| Men 0 vs 1.5 | ns | ns | ns |
| Men 0 vs 2.0 | ns | ns | ns |
| Men 0 vs 2.5 | ns | ns | ns |
| Men 0 vs 3.0 | ns | ns | ns |
| Men 0 vs 3.5 | ns | ns | ns |

|  | E-score | S-score | T-score |
| --- | --- | --- | --- |
|  | Femoris | Femoris | Femoris |
| Women 0 vs 0.5 | ns | ns | ns |
| Women 0 vs 1.0 | ns | ns | ns |
| Women 0 vs 1.5 | ns | ns | ns |
| Women 0 vs 2.0 | ns | ns | ns |
| Women 0 vs 2.5 | ns | ns | ns |
| Women 0 vs 3.0 | ns | ns | ns |
| Women 0 vs 3.5 | ns | ns | ns |

Age

| Load 0 | E-score | S-score | T-score |
| --- | --- | --- | --- |
|  | Femoris | Femoris | Femoris |
| Men 20 vs 30 | ns | ns | ns |
| Men 30 vs 40 | ns | ns | ns |
| Men 40 vs 50 | ns | ns | ns |
| Men 50 vs 60 | ns | ns | ns |
| Men 20 vs 40 | ns | ns | ns |
| Men 20 vs 50 | ns | ns | ns |
| Men 20 vs 60 | ns | ns | ns |
| Men 30 vs 50 | ns | ns | ns |
| Men 30 vs 60 | ns | ns | ns |
| Men 40 vs 60 | ns | ns | ns |

| Load 0.5 | E-score | S-score | T-score |
| --- | --- | --- | --- |
|  | Femoris | Femoris | Femoris |
| Men 20 vs 30 | ns | ns | ns |
| Men 30 vs 40 | ns | ns | ns |
| Men 40 vs 50 | ns | ns | ns |
| Men 50 vs 60 | ns | ns | ns |
| Men 20 vs 40 | ns | ns | ns |
| Men 20 vs 50 | ns | ns | ns |
| Men 20 vs 60 | ns | ns | ns |
| Men 30 vs 50 | ns | ns | ns |
| Men 30 vs 60 | ns | ns | ns |
| Men 40 vs 60 | ns | ns | ns |

| Load 1.0 | E-score | S-score | T-score |
| --- | --- | --- | --- |
|  | Femoris | Femoris | Femoris |
| Men 20 vs 30 | ns | ns | ns |
| Men 30 vs 40 | ns | ns | ns |
| Men 40 vs 50 | ns | ns | ns |
| Men 50 vs 60 | ns | ns | ns |
| Men 20 vs 40 | ns | ns | ns |
| Men 20 vs 50 | ns | ns | ns |
| Men 20 vs 60 | ns | ns | ns |
| Men 30 vs 50 | ns | ns | ns |
| Men 30 vs 60 | ns | ns | ns |
| Men 40 vs 60 | ns | ns | ns |

| Load 1.5 | E-score | S-score | T-score |
| --- | --- | --- | --- |
|  | Femoris | Femoris | Femoris |
| Men 20 vs 30 | ns | ns | ns |
| Men 30 vs 40 | ns | ns | ns |
| Men 40 vs 50 | ns | ns | ns |
| Men 50 vs 60 | ns | ns | ns |
| Men 20 vs 40 | ns | ns | ns |
| Men 20 vs 50 | ns | ns | ns |
| Men 20 vs 60 | ns | ns | ns |
| Men 30 vs 50 | ns | ns | ns |
| Men 30 vs 60 | ns | ns | ns |
| Men 40 vs 60 | ns | ns | ns |

| Load 2.0 | E-score | S-score | T-score |
| --- | --- | --- | --- |
|  | Femoris | Femoris | Femoris |
| Men 20 vs 30 | ns | ns | ns |
| Men 30 vs 40 | ns | ns | ns |
| Men 40 vs 50 | ns | ns | ns |
| Men 50 vs 60 | ns | ns | ns |
| Men 20 vs 40 | ns | ns | ns |
| Men 20 vs 50 | ns | ns | ns |
| Men 20 vs 60 | ns | ns | ns |
| Men 30 vs 50 | ns | ns | ns |
| Men 30 vs 60 | ns | ns | ns |
| Men 40 vs 60 | ns | ns | ns |

| Load 2.5 | E-score | S-score | T-score |
| --- | --- | --- | --- |
|  | Femoris | Femoris | Femoris |
| Men 20 vs 30 | ns | ns | ns |
| Men 30 vs 40 | ns | ns | ns |
| Men 40 vs 50 | ns | ns | ns |
| Men 50 vs 60 | ns | ns | ns |
| Men 20 vs 40 | ns | ns | ns |
| Men 20 vs 50 | ns | ns | ns |
| Men 20 vs 60 | ns | ns | ns |
| Men 30 vs 50 | ns | ns | ns |
| Men 30 vs 60 | ns | ns | ns |
| Men 40 vs 60 | ns | ns | ns |

| Load 3.0 | E-score | S-score | T-score |
| --- | --- | --- | --- |
|  | Femoris | Femoris | Femoris |
| Men 20 vs 30 | ns | ns | ns |
| Men 30 vs 40 | ns | ns | ns |
| Men 40 vs 50 | ns | ns | ns |
| Men 50 vs 60 | ns | ns | ns |
| Men 20 vs 40 | ns | ns | ns |
| Men 20 vs 50 | ns | ns | ns |
| Men 20 vs 60 | ns | ns | ns |
| Men 30 vs 50 | ns | ns | ns |
| Men 30 vs 60 | ns | ns | ns |
| Men 40 vs 60 | ns | ns | ns |

| Load 3.5 | E-score | S-score | T-score |
| --- | --- | --- | --- |
|  | Femoris | Femoris | Femoris |
| Men 20 vs 30 | ns | ns | ns |
| Men 30 vs 40 | ns | ns | ns |
| Men 40 vs 50 | ns | ns | ns |
| Men 50 vs 60 | ns | ns | ns |
| Men 20 vs 40 | ns | ns | ns |
| Men 20 vs 50 | ns | ns | ns |
| Men 20 vs 60 | ns | ns | ns |
| Men 30 vs 50 | ns | ns | ns |
| Men 30 vs 60 | ns | ns | ns |
| Men 40 vs 60 | ns | ns | ns |

| Load 0 | E-score | S-score | T-score |
| --- | --- | --- | --- |
|  | Femoris | Femoris | Femoris |
| Women 20 vs 30 | ns | ns | ns |
| Women 30 vs 40 | ns | ns | ns |
| Women 40 vs 50 | ns | ns | ns |
| Women 50 vs 60 | ** | ns | ns |
| Women 20 vs 40 | ns | ns | ns |
| Women 20 vs 50 | ns | ns | ns |
| Women 20 vs 60 | ns | ns | ns |
| Women 30 vs 50 | ns | ns | ns |
| Women 30 vs 60 | ns | ns | ns |
| Women 40 vs 60 | ns | ns | ns |

| Load 0.5 | E-score | S-score | T-score |
| --- | --- | --- | --- |
|  | Femoris | Femoris | Femoris |
| Women 20 vs 30 | ns | ns | ns |
| Women 30 vs 40 | ns | ns | ns |
| Women 40 vs 50 | ns | ns | ns |
| Women 50 vs 60 | *** | ns | ns |
| Women 20 vs 40 | ns | ns | ns |
| Women 20 vs 50 | ns | ns | ns |
| Women 20 vs 60 | ns | ns | ns |
| Women 30 vs 50 | ns | ns | ns |
| Women 30 vs 60 | ns | ns | ns |
| Women 40 vs 60 | ns | ns | ns |

| Load 1.0 | E-score | S-score | T-score |
| --- | --- | --- | --- |
|  | Femoris | Femoris | Femoris |
| Women 20 vs 30 | ns | ns | ns |
| Women 30 vs 40 | ns | ns | ns |
| Women 40 vs 50 | ns | ns | ns |
| Women 50 vs 60 | *** | ns | ns |
| Women 20 vs 40 | ns | ns | ns |
| Women 20 vs 50 | ns | ns | ns |
| Women 20 vs 60 | ns | ns | ns |
| Women 30 vs 50 | ns | ns | ns |
| Women 30 vs 60 | ns | ns | ns |
| Women 40 vs 60 | ns | ns | ns |

| Load 1.5 | E-score | S-score | T-score |
| --- | --- | --- | --- |
|  | Femoris | Femoris | Femoris |
| Women 20 vs 30 | ns | ns | ns |
| Women 30 vs 40 | ns | ns | ns |
| Women 40 vs 50 | ns | ns | ns |
| Women 50 vs 60 | *** | ns | ns |
| Women 20 vs 40 | ns | ns | ns |
| Women 20 vs 50 | ns | ns | ns |
| Women 20 vs 60 | ns | ns | ns |
| Women 30 vs 50 | ns | ns | ns |
| Women 30 vs 60 | ns | ns | ns |
| Women 40 vs 60 | ns | ns | ns |

| Load 2.0 | E-score | S-score | T-score |
| --- | --- | --- | --- |
|  | Femoris | Femoris | Femoris |
| Women 20 vs 30 | ns | ns | ns |
| Women 30 vs 40 | ns | ns | ns |
| Women 40 vs 50 | ns | ns | ns |
| Women 50 vs 60 | *** | ns | ns |
| Women 20 vs 40 | ns | ns | ns |
| Women 20 vs 50 | ns | ns | ns |
| Women 20 vs 60 | ns | ns | ns |
| Women 30 vs 50 | ns | ns | ns |
| Women 30 vs 60 | ns | ns | ns |
| Women 40 vs 60 | ns | ns | ns |

| Load 2.5 | E-score | S-score | T-score |
| --- | --- | --- | --- |
|  | Femoris | Femoris | Femoris |
| Women 20 vs 30 | ns | ns | ns |
| Women 30 vs 40 | ns | ns | ns |
| Women 40 vs 50 | *** | ns | ns |
| Women 50 vs 60 | *** | ns | ns |
| Women 20 vs 40 | ns | ns | ns |
| Women 20 vs 50 | ns | ns | ns |
| Women 20 vs 60 | ns | ns | ns |
| Women 30 vs 50 | ** | ns | ns |
| Women 30 vs 60 | ns | ns | ns |
| Women 40 vs 60 | ns | ns | ns |

| Load 3.0 | E-score | S-score | T-score |
| --- | --- | --- | --- |
|  | Femoris | Femoris | Femoris |
| Women 20 vs 30 | ns | ns | ns |
| Women 30 vs 40 | ns | ns | ns |
| Women 40 vs 50 | *** | ns | ns |
| Women 50 vs 60 | *** | ns | ns |
| Women 20 vs 40 | ns | ns | ns |
| Women 20 vs 50 | ns | ns | ns |
| Women 20 vs 60 | ns | ns | ns |
| Women 30 vs 50 | ** | ns | ns |
| Women 30 vs 60 | ns | ns | ns |
| Women 40 vs 60 | ns | ns | ns |

| Load 3.5 | E-score | S-score | T-score |
| --- | --- | --- | --- |
|  | Femoris | Femoris | Femoris |
| Women 20 vs 30 | ns | ns | ns |
| Women 30 vs 40 | ns | ns | ns |
| Women 40 vs 50 | ** | ns | ns |
| Women 50 vs 60 | *** | ns | ** |
| Women 20 vs 40 | ns | ns | ns |
| Women 20 vs 50 | ns | ns | ns |
| Women 20 vs 60 | ns | ns | ns |
| Women 30 vs 50 | ns | ns | ns |
| Women 30 vs 60 | ns | ns | ns |
| Women 40 vs 60 | ns | ns | ns |

ARM FLEXION – EXTENSION

Biceps

Passive

Men versus Women

|  | E-score | S-score | T-score |
| --- | --- | --- | --- |
| Men vs Women Biceps | ns | ns | ns |
|  |  |  |  |
|  |  |  |  |

Age differences

(1-5 refers to the different age groups)

|  | E-score | S-score | T-score |
| --- | --- | --- | --- |
| Men Biceps |  |  |  |
| 1-2 | ns | ns | ns |
| 2-3 | ns | ns | ns |
| 3-4 | ns | ns | ns |
| 4-5 | *** | ns | ns |
| 1-3 | ns | ns | ns |
| 1-4 | ns | ns | ns |
| 1-5 | *** | ns | ns |
| 2-4 | ns | ns | ns |
| 2-5 | *** | ns | ns |
| 3-5 | *** | ns | ns |
|  |  |  |  |

|  | E-score | S-score | T-score |
| --- | --- | --- | --- |
| Women Biceps |  |  |  |
| 1-2 | ns | ns | ns |
| 2-3 | ns | ns | ns |
| 3-4 | ns | ns | ns |
| 4-5 | *** | ns | ns |
| 1-3 | ns | ns | ns |
| 1-4 | ns | ns | ns |
| 1-5 | * | ns | ns |
| 2-4 | ns | ns | ns |
| 2-5 | ns | ns | ns |
| 3-5 | ns | ns | ns |
|  |  |  |  |

Active

Men versus Women

|  | E-score | S-score | T-score |
| --- | --- | --- | --- |
| Men vs Women Biceps | ns | ns | ns |
|  |  |  |  |
|  |  |  |  |

Age differences

(1-5 refers to the different age groups)

|  | E-score | S-score | T-score |
| --- | --- | --- | --- |
| Men Biceps |  |  |  |
| 1-2 | ns | ns | ns |
| 2-3 | ns | ns | ns |
| 3-4 | ns | ns | ns |
| 4-5 | ns | ns | ns |
| 1-3 | ns | ns | ns |
| 1-4 | ns | ns | ns |
| 1-5 | * |  |  |
| 2-4 | ns | ns | ns |
| 2-5 | * |  |  |
| 3-5 | ns | ns | ns |
|  |  |  |  |

|  | E-score | S-score | T-score |
| --- | --- | --- | --- |
| Women Biceps |  |  |  |
| 1-2 | ns | ns | ns |
| 2-3 | ns | ns | ns |
| 3-4 | ns | ns | ns |
| 4-5 | ns | ns | ns |
| 1-3 | ns | ns | ns |
| 1-4 | ns | ns | ns |
| 1-5 | ns | ns | ns |
| 2-4 | ns | ns | ns |
| 2-5 | ns | ns | ns |
| 3-5 | ns | ns | ns |
|  |  |  |  |

Triceps

Passive

Men versus Women

|  | E-score | S-score | T-score |
| --- | --- | --- | --- |
| Men vs Women Triceps | ns | ns | ns |
|  |  |  |  |
|  |  |  |  |

Age differences

(1-5 refers to the different age groups)

|  | E-score | S-score | T-score |
| --- | --- | --- | --- |
| Men Triceps |  |  |  |
| 1-2 | ns | ns | ns |
| 2-3 | ns | ns | ns |
| 3-4 | ns | ns | ns |
| 4-5 | * | ns | ns |
| 1-3 | ns | ns | ns |
| 1-4 | ns | ns | ns |
| 1-5 | ns | ns | ns |
| 2-4 | ns | ns | ns |
| 2-5 | ns | ns | ns |
| 3-5 | ns | ns | ns |
|  |  |  |  |

|  | E-score | S-score | T-score |
| --- | --- | --- | --- |
| Women Triceps |  |  |  |
| 1-2 | ns | ns | ns |
| 2-3 | ns | ns | ns |
| 3-4 | ns | ns | ns |
| 4-5 | * | ns | ns |
| 1-3 | ns | ns | ns |
| 1-4 | ns | ns | ns |
| 1-5 | ns | ns | ns |
| 2-4 | ns | ns | ns |
| 2-5 | ns | ns | ns |
| 3-5 | ns | ns | ns |
|  |  |  |  |

Active

Men versus Women

|  | E-score | S-score | T-score |
| --- | --- | --- | --- |
| Men vs Women Triceps | ns | ns | ns |
|  |  |  |  |
|  |  |  |  |

Age differences

(1-5 refers to the different age groups)

|  | E-score | S-score | T-score |
| --- | --- | --- | --- |
| Men Triceps |  |  |  |
| 1-2 | ns | ns | ns |
| 2-3 | ns | ns | ns |
| 3-4 | ns | ns | ns |
| 4-5 | ns | ns | ns |
| 1-3 | ns | ns | ns |
| 1-4 | ns | ns | ns |
| 1-5 | ns | ns | ns |
| 2-4 | ns | ns | ns |
| 2-5 | ns | ns | * |
| 3-5 | ns | ns | ns |
|  |  |  |  |

|  | E-score | S-score | T-score |
| --- | --- | --- | --- |
| Women Triceps |  |  |  |
| 1-2 | ns | ns | ns |
| 2-3 | ns | ns | ns |
| 3-4 | ns | ns | ns |
| 4-5 | * | ns | ns |
| 1-3 | ns | ns | ns |
| 1-4 | ns | ns | ns |
| 1-5 | * | ns | ns |
| 2-4 | ns | ns | ns |
| 2-5 | ns | ns | ns |
| 3-5 | ns | ns | ns |
|  |  |  |  |

Active versus Passive - Biceps & Triceps

| Biceps | E-score | S-score | T-score |
| --- | --- | --- | --- |
| Men active vs passive | ns | ns | ns |
| Women active vs passive | ns | ns | ns |
|  |  |  |  |
|  |  |  |  |

| Triceps | E-score | S-score | T-score |
| --- | --- | --- | --- |
| Men active vs passive | ns | ns | ns |
| Women active vs passive | ns | ns | ns |
|  |  |  |  |
|  |  |  |  |

WRITING TEST - Trapezius

Men versus Women

|  | E-score | S-score | T-score |
| --- | --- | --- | --- |
|  | Left / Right | Left / Right | Left / Right |
| Men vs Women | ns / ns | ns / ns | ns / ns |
|  |  |  |  |
|  |  |  |  |

Left versus Right

|  | E-score | S-score | T-score |
| --- | --- | --- | --- |
| Men left vs right | ns | ns | ns |
| Women left vs right | ns | ns | ns |
|  |  |  |  |
|  |  |  |  |

An ANOVA of rows shows no significant age effect for any of the parameters

THUMB TEST – Abductor pollicis

Men versus Women – Flexion/Extension & Touching Little Finger with Thumb

|  | E-score | S-score | T-score |
| --- | --- | --- | --- |
|  | Left / Right | Left / Right | Left / Right |
| Men vs Women flex | ns | ns | ns |
| Men vs Women little | ns | ns | ns |
|  |  |  |  |

An ANOVA of rows shows no significant age effect for any of the parameters
